# Disruption of the Putative Ribosome-Binding Motif of a Scaffold Protein Impairs Cytochrome *c* Oxidase Subunit Expression in *Leishmania major*

**DOI:** 10.1128/mSphere.00644-18

**Published:** 2019-03-06

**Authors:** Daviel Cardenas, Charity Sylvester, Bo Cao, Catherine S. Nation, Juan C. Pizarro, Hua Lu, Jessie Guidry, Edward J. Wojcik, Ben L. Kelly

**Affiliations:** a Department of Microbiology, Immunology and Parasitology, Louisiana State University Health Sciences Center, New Orleans, Louisiana, USA; b Department of Biochemistry & Molecular Biology and Tulane Cancer Center, Tulane University School of Medicine, New Orleans, Louisiana, USA; c Department of Tropical Medicine, Tulane University, New Orleans, Louisiana, USA; d Department of Biochemistry & Molecular Biology, Louisiana State University Health Sciences Center, New Orleans, Louisiana, USA; University of Georgia

**Keywords:** LACK, *Leishmania*, RACK1, cytochrome *c* oxidase, mitochondria, parasite, ribosome, translation

## Abstract

During their parasitic life cycle, through sandflies and vertebrate hosts, Leishmania parasites confront strikingly different environments, including abrupt changes in pH and temperature, to which they must rapidly adapt. These adaptations include alterations in *Leishmania* gene expression, metabolism, and morphology, allowing them to thrive as promastigotes in the sandfly and as intracellular amastigotes in the vertebrate host. A critical aspect of *Leishmania* metabolic adaptation to these changes is maintenance of efficient mitochondrial function in the hostile vertebrate environment. Such functions, including generation of ATP, depend upon the expression of many mitochondrial proteins, including subunits of cytochrome *c* oxidase (COX). Significantly, under mammalian temperature conditions, expression of Leishmania major COX subunit IV (LmCOX4) and virulence are dependent upon two copies of *LACK*, a gene that encodes the ribosome-associated scaffold protein, LACK (*Leishmania* ortholog of RACK1 [receptor for activated C kinase]). Targeted replacement of an endogenous *LACK* copy with a putative ribosome-binding motif-disrupted variant (LACK^R34D35G36^→LACK^D34D35E36^) resulted in thermosensitive parasites that showed diminished LmCOX4 expression, mitochondrial fitness, and replication in macrophages. Surprisingly, despite these phenotypes, LACK^D34D35E36^ associated with monosomes and polysomes and showed no major impairment of global protein synthesis. Collectively, these data suggest that wild-type (WT) LACK orchestrates robust LmCOX4 expression and mitochondrial fitness to ensure parasite virulence, via optimized functional interactions with the ribosome.

**IMPORTANCE** *Leishmania* parasites are trypanosomatid protozoans that persist in infected human hosts to cause a spectrum of pathologies, from cutaneous and mucocutaneous manifestations to visceral leishmaniasis caused by Leishmania donovani. The latter is usually fatal if not treated. Persistence of L. major in the mammalian host depends upon maintaining gene-regulatory programs to support essential parasite metabolic functions. These include expression and assembly of mitochondrial *L. major* cytochrome *c* oxidase (LmCOX) subunits, important for *Leishmania* ATP production. Significantly, under mammalian conditions, WT levels of LmCOX subunits require threshold levels of the *Leishmania* ribosome-associated scaffold protein, LACK. Unexpectedly, we find that although disruption of LACK’s putative ribosome-binding motif does not grossly perturb ribosome association or global protein synthesis, it nonetheless impairs COX subunit expression, mitochondrial function, and virulence. Our data indicate that the quality of LACK’s interaction with *Leishmania* ribosomes is critical for LmCOX subunit expression and parasite mitochondrial function in the mammalian host. Collectively, these findings validate LACK’s ribosomal interactions as a potential therapeutic target.

## Abstract (precis)

*Leishmania* parasites are trypanosomatid protozoans that persist in infected human hosts to cause a spectrum of pathologies, from cutaneous and mucocutaneous manifestations to visceral leishmaniasis caused by Leishmania donovani. The latter is usually fatal if not treated. Persistence of L. major in the mammalian host depends upon maintaining gene-regulatory programs to support essential parasite metabolic functions. These include expression and assembly of mitochondrial *L. major* cytochrome *c* oxidase (LmCOX) subunits, important for *Leishmania* ATP production. Significantly, under mammalian conditions, WT levels of LmCOX subunits require threshold levels of the *Leishmania* ribosome-associated scaffold protein, LACK. Unexpectedly, we find that although disruption of LACK’s putative ribosome-binding motif does not grossly perturb ribosome association or global protein synthesis, it nonetheless impairs COX subunit expression, mitochondrial function, and virulence. Our data indicate that the quality of LACK’s interaction with *Leishmania* ribosomes is critical for LmCOX subunit expression and parasite mitochondrial function in the mammalian host. Collectively, these findings validate LACK’s ribosomal interactions as a potential therapeutic target.

## INTRODUCTION

Leishmania species are dimorphic parasites that exist as flagellated, extracellular promastigotes in the alimentary canal of sandflies and as nonflagellated, intracellular amastigotes in the phagolysosome of vertebrate host macrophages. Compared to the sandfly gut environment, the mammalian macrophage phagolysosome niche has elevated temperature, acidic pH, and decreased glucose concentration (1). In response to the abrupt environmental changes encountered during transition from the sandfly to the mammalian host, *Leishmania* parasites undergo important alterations in gene expression (2). These changes result in appropriate metabolic and morphological adaptations to ensure survival and replication in the vertebrate host (1). Because *Leishmania* gene expression is controlled largely via posttranscriptional mechanisms, translational regulatory mechanisms are considered particularly important for this trypanosomatid (3, 4). Despite their importance, however, our understanding of *Leishmania* molecular mechanisms that regulate protein expression at elevated temperature remains limited.

We previously identified LACK, a ribosome-associated member of the multifunctional RACK1 family of WD40 scaffold proteins, as a factor important for Leishmania major thermotolerance and hence virulence in the mammalian host (5). Recently, we determined that threshold levels of LACK expressed from a minimum of two *LACK* copies are required for maintaining L. major cytochrome *c* oxidase subunit IV (LmCOX4) levels, mitochondrial fitness, and thermotolerance (6). LmCOX4 is a subunit of cytochrome *c* oxidase (LmCOX), an important protein complex (termed complex IV) within the electron transport chain of the inner mitochondrial membrane. This complex is critical for generating the mitochondrial proton gradient for cellular ATP production.

Earlier, we demonstrated that, similar to other RACK1 family members, LACK has translation-associated functions (7). In mammalian and yeast cells, previous studies indicate that the association of RACK1 with the 40S ribosomal subunit occurs via a net-positively-charged three-amino-acid motif, RDK, within the first WD40 domain (8, 9). Indeed, in Saccharomyces cerevisiae, substitution of this motif for a negatively charged DDE element disrupted RACK1’s association with polysomes (8). Based on these studies, the RACK1^DDE^ variant has therefore been used to dissect the ribosome-associated functions of RACK1s from their other functions (8, 10). The RDK motif is highly conserved among eukaryote RACK1s, including those from Homo sapiens, Saccharomyces cerevisiae, Cryptococcus neoformans, and the trypanosomatid protozoan parasite Trypanosoma brucei (9, 11). Intriguingly, despite the close evolutionary relatedness of T. brucei and L. major, the orthologous motif in LACK is RDG.

To gain insight into potential translation-associated mechanisms that may underlie LACK-dependent expression of LmCOX4 at mammalian temperature, we used a DDE mutant substitution approach intended to perturb LACK’s interaction with *Leishmania* ribosomes. We then assessed the ability of LACK^DDE^ to associate with polysomes and determined the effect of *LACK^DDE^* gene replacement upon global protein synthesis, LACK-dependent modulation of LmCOX4, mitochondrial fitness, and infectivity.

We employed a gene-targeting strategy in which the endogenous downstream *LACK* copy of *LACK*/*LACK* L. major was replaced with a hemagglutinin (HA)-tagged variant of *LACK* encoding the DDE variant motif, to generate the *LACK*/*LACK^DDE^* L. major line. Another line, designated *LACK/LACK^RDG^* L. major, expressing a similarly targeted HA-tagged wild-type (WT) *LACK*, was used as a control (7).

We found that despite the phenotypic defects observed in the *LACK*/*LACK^DDE^* line, LACK^DDE^ associated robustly with polysomes. Interestingly, although altered polysome profiles were observed, global protein synthesis in this line was unimpaired. The defective phenotypes associated with *LACK^DDE^* substitution suggest LACK’s importance in LmCOX subunit expression, mitochondrial fitness, and virulence is linked to the quality of its ribosomal interactions through its RDG motif.

## RESULTS

### Generation of LACK ribosome-binding motif ortholog mutants.

*LACK-*haploinsufficient parasites, termed *LACK*/− (6), are virulence attenuated and highly sensitive to the translation initiation inhibitor hippuristanol compared to WT controls (7). These findings suggest LACK is a functionally important constituent of *Leishmania* translation initiation. In pursuit of LACK’s molecular function in virulence, we recently determined that threshold levels of LACK are required for maintenance of LmCOX4 and LmCOX6 expression and mitochondrial function under mammalian conditions (6). Molecular mechanisms linking LACK’s translation-associated functions with LmCOX4 expression and virulence, however, have not previously been investigated.

Earlier studies (8, 9) identified a conserved three-amino-acid motif, R_38_D_39_K_40_, within WD40 domain 1 of most RACK1 family members. This motif has been described to mediate RACK1 association with the ribosome, where the two positively charged amino acids in the motif were substituted for negatively charged residues (RDK→DDE). Importantly, these substitutions can result in mutant RACK1 proteins with decreased ribosome binding properties and impaired translational functions (10, 12).

**FIG 1:**
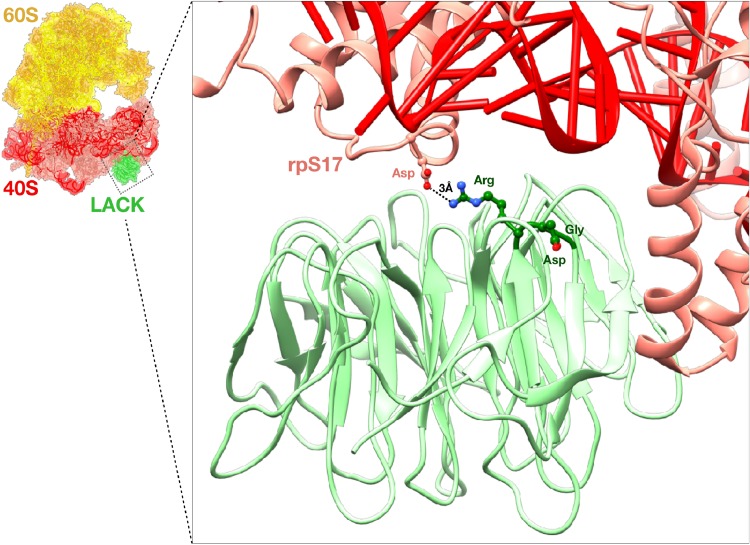
Structural model of the interaction between LACK and the *Leishmania* ribosome. The structure of a *Leishmania* ribosome (60S and 40S subunits are indicated in yellow and red, respectively) from Zhang et al. (13), modeled in association with LACK (green), is shown. The inset shows detail of the predicted electrostatic interaction between amino acid residue Arg_34_ of LACK’s RDG motif and D_27_ of rpS17 of the 40S ribosomal subunit. RDG residues D_35_ and G_36_ are also indicated.

Based on amino acid sequence alignment between T. brucei RACK1 (TbRACK1) and LACK, we identified a partially divergent three-amino-acid sequence in LACK, R_34_D_35_G_36_, as the *Leishmania* ortholog of the RDK motif (7) (see Fig. S1 in the supplemental material). Despite LACK possessing a noncanonical RDG motif, previous studies indicate that LACK associates with L. major ribosomes (7). Furthermore, analysis of LACK’s interaction with *Leishmania* ribosomes (13) predicted that only R_34_ of this motif interacts productively, at a distance of 3 Å, with D_27_ of the 40S ribosomal subunit protein, rpS17 (Fig. 1). In light of these observations, we reasoned that substitution for LACK’s R_34_D_35_G_36_ motif with the established DDE mutant sequence would impair its functional association with *Leishmania* ribosomes. Therefore, if LACK promotes LmCOX subunit expression via its ribosome-associated functions, we would expect substitution of an endogenous WT *LACK* copy for *LACK^DDE^* to impair LmCOX subunit expression and parasite fitness.

10.1128/mSphere.00644-18.1 FIG S1 Sequence alignments to pinpoint RDG motif. Alignment of the amino acid sequences for L. major LACK and TbRACK1 was performed using BLAST (bl2seq; https://blast.ncbi.nlm.nih.gov/Blast.cgi?PAGE=Proteins&PROGRAM=blastp&BLAST_PROGRAMS=blastp&PAGE_TYPE=BlastSearch&BLAST_SPEC=blast2seq). Identical conserved amino acids between LACK and TbRACK1 are denoted by the single-letter code. Similar amino acids are indicated by a plus sign. The putative ribosome-binding motif R_34_D_35_G_36_ is indicated in boldface. Download FIG S1, PDF file, 0.07 MB. Copyright © 2019 Cardenas et al. 2019 Cardenas et al. This content is distributed under the terms of the Creative Commons Attribution 4.0 International license.

**FIG 2:**
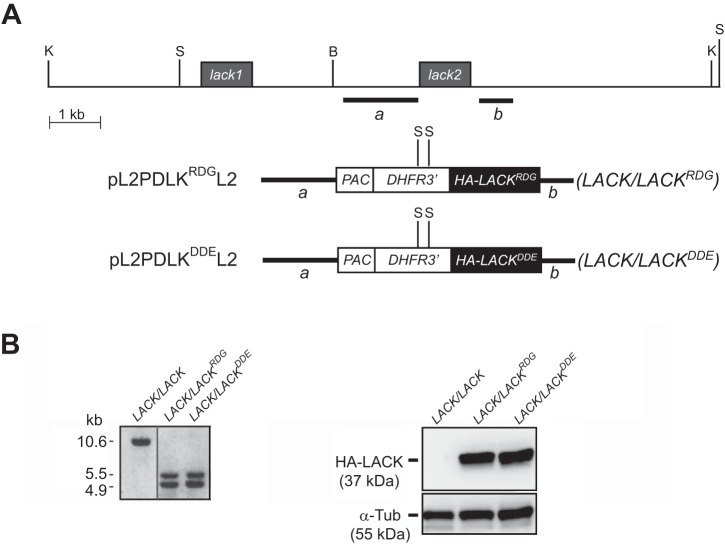
Creation of the *LACK*/*LACK^DDE^* and *LACK*/*LACK^RDG^* L. major lines. (A) Schematic of the *LACK* gene-targeting strategy. The 5′ (*a*) and 3′ (*b*) targeting flanks of *LACK2* are indicated. “S” and “K” denote the StuI and KpnI restriction endonuclease sites, respectively, flanking the endogenous *LACK* genes of L. major and StuI sites located in the *DHFR3′* sequence of the *PAC*/*DHFR3′* expression cassette. “B” denotes a BamHI restriction endonuclease site. (B, left panel) Southern blot analysis showing gene fragments of the anticipated lengths, for each line, as indicated. Left lane: virulent parental line (*LACK*/*LACK*) (6), with two endogenous copies of *LACK* (*lack1* and *lack2*). Middle lane: representative clone of the *LACK*/*LACK^RDG^* line, with one upstream WT endogenous *LACK* copy (*lack1*) and targeted replacement of the downstream *LACK* gene (*lack2*) with HA-tagged WT *LACK* (*LACK^RDG^*). Right lane: a representative clone of the *LACK*/*LACK^DDE^* line, with one upstream WT endogenous *LACK* copy (*lack1*) and targeted replacement of the downstream *LACK* (*lack2*) with HA-tagged LACK-D_34_D_35_E_36_ mutant (*LACK^DDE^*). Genomic DNAs were digested with StuI, and hybridization was performed using the labeled *LACK* ORF as a DNA probe. (B, right panel) Immunoblot analysis using anti-HA antibody showing expression of an HA-tagged protein of the expected sizes in the *LACK*/*LACK^RDG^* and *LACK*/*LACK^DDE^* lines (upper section, middle and right lanes, respectively) as indicated. Expression of α-tubulin (α-Tub) for each line (lower section) is denoted.

We used site-directed mutagenesis of the *LACK* open reading frame (ORF) to substitute the RDG amino acid sequence motif (residues 34 to 36) for DDE. Our gene-targeting approach, as indicated in Fig. 2A, was to replace the second *LACK* copy (*LACK2*) of *LACK*/*LACK* L. major with an HA-tagged *LACK* ORF encoding the D_34_D_35_E_36_ mutant motif. This *LACK*-targeting strategy has been described previously to demonstrate the threshold requirement for two *LACK* copies for robust parasite fitness and LmCOX subunit expression under mammalian conditions (6, 7). We also created an isogenic control strain in which *LACK2* of *LACK*/*LACK* L. major was replaced with an HA-tagged copy of WT *LACK*, encoding the R_34_D_35_G_36_ motif as previously described (7). Following electroporation, transfectant lines were selected by plating with puromycin and successful targeting was confirmed by Southern blot analysis (Fig. 2B, left panel), as previously described (7). Comparable DNA loading in each line was confirmed (see Fig. S2 in the supplemental material). We then confirmed expression of HA-LACK in these cloned lines using an anti-HA antibody (Fig. 2B, right panel). These transfectants, each expressing an HA-tagged protein of the expected size, were designated *LACK*/*LACK^DDE^* and *LACK*/*LACK^RDG^*, respectively.

10.1128/mSphere.00644-18.2 FIG S2 Genomic DNA ethidium bromide (loading) gel. Shown is the ethidium bromide-stained agarose gel of fractionated StuI genomic fragments, showing similar DNA loading for L. major lines. Download FIG S2, PDF file, 0.04 MB. Copyright © 2019 Cardenas et al. 2019 Cardenas et al. This content is distributed under the terms of the Creative Commons Attribution 4.0 International license.

### Fitness of *LACK*/*LACK^DDE^ L. major* is decreased at 35°C.

**FIG 3:**
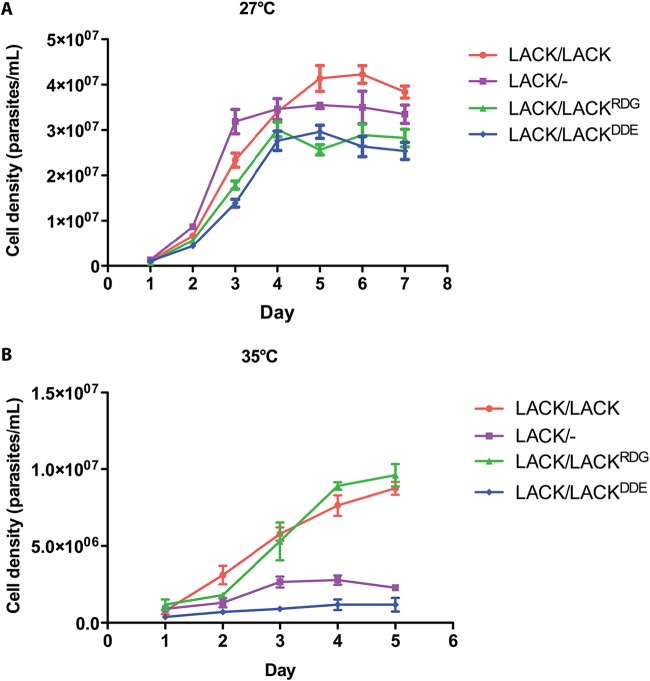
The *LACK*/*LACK^DDE^* line displays attenuated growth at 35°C. (A) The *LACK*/*LACK*, *LACK*/−, *LACK*/*LACK^RDG^*, and *LACK*/*LACK^DDE^* parasite lines, as indicated, were seeded into medium 199 with 10% FBS, as described in Materials and Methods, at a density of 3 × 10^5^ promastigotes/ml. Cell densities were determined daily for 7 days at 27°C using a hemocytometer to enumerate parasites fixed in 0.4% formaldehyde–PBS. (B) Parasite lines, as described in panel A, seeded at 2 × 10^6^ promastigotes/ml, were incubated overnight at 35°C, diluted 1:10, and then incubated for 5 additional days at 35°C. Cell densities were assessed daily, as in panel A. Data are shown as mean ± standard error of the mean (SEM [indicated by error bars]) from experiments with two independent clones of each line, each performed in duplicate.

*LACK*/*LACK^DDE^*, *LACK*/*LACK^RDG^*, *LACK*/*LACK*, and *LACK*/− L. major parasites all showed comparable viabilities at 27°C, as indicated by their similar growth profiles (Fig. 3A) and the similar proportions of live cells within each culture (see Fig. S3 in the supplemental material). Conversely, at 35°C, *LACK*/*LACK^RDG^* and *LACK*/*LACK* parasites showed robust viability, whereas the viability of *LACK*/*LACK^DDE^* and *LACK*/− L. major was markedly decreased (Fig. 3B).

10.1128/mSphere.00644-18.3 FIG S3 *LACK*/*LACK, LACK*/−, *LACK*/*LACK^RDG^*, and *LACK*/*LACK^DDE^* L. major cultures show similar live (fluorescein diacetate [FDA]-positive) cell populations of cells. Parasite lines, as indicated, were cultured for 4 days at 27°C and then diluted 1/10 in PBS and enumerated by hemocytometer. After normalizing for cell population density, equal numbers of parasites were stained with FDA, and then FDA-positive cells were counted by fluorescence microscopy as previously described (6). “HK” denotes *LACK*/*LACK* L. major parasites that were heat killed by incubation at 75°C for 10 min prior to FDA staining. Download FIG S3, PDF file, 0.05 MB. Copyright © 2019 Cardenas et al. 2019 Cardenas et al. This content is distributed under the terms of the Creative Commons Attribution 4.0 International license.

### LACK^DDE^ associates efficiently with *Leishmania* ribosomes at 27 and 35°C.

**FIG 4:**
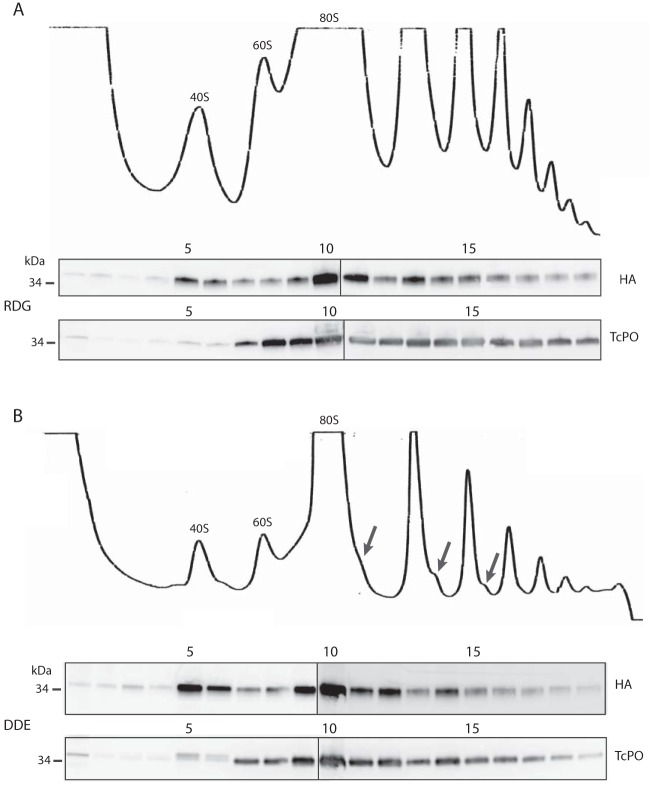
*LACK^DDE^* associates with polysomes at 27°C. Lysates from the logarithmically growing *LACK*/*LACK^RDG^* (RDG) and *LACK*/*LACK^DDE^* (DDE) lines at 27°C were prepared for sucrose gradient fractionation as described in Materials and Methods and then applied to a linear 10 to 50% sucrose gradient and centrifuged for 120 min at 35,000 rpm. After rRNA profiling (optical density at 254 nm [OD_254_]) and fractionation of the gradient into 0.5-ml aliquots, proteins were isolated from each fraction by methanol-chloroform precipitation. The fractions were separated on SDS-PAGE gels and probed with anti-HA and anti-TcP0 antisera, as indicated. The polysome profiles are shown at the top of each panel for LACK^RDG^ (upper panel) and LACK^DDE^ (lower panel), respectively, as indicated. The top of the sucrose gradient corresponds to the left side of the panels. Black arrows denote ribosome/polysome half-mers observed in the *LACK*/*LACK^DDE^* line (lower panel). “40S,” “60S,” and “80S” denote the 40S and 60S ribosomal subunits and 80S monosomes, respectively. Fraction numbers are indicated above each blot. The data shown are from one experiment, representative of experiments with two independent clones, each performed in duplicate. Protein size markers (kDa) are indicated to the left of each panel.

*LACK/LACK^RDG^* L. major, growing logarithmically at 27°C, showed a typical polysome profile (Fig. 4A), indicating robustly translating ribosomes, similar to previous studies (14). Consistent with other RACK1 family members (15), WT LACK also shows preferential cosedimentation with 40S subunit fractions (Fig. 4A, fractions 5 and 6) compared to the free 60S subunit fractions (Fig. 4A, fractions 7 to 8). Conversely, P0 is a component of the 60S ribosome (16) and, as expected, is enriched in all fractions containing the 60S subunit (Fig. 4A, fraction 7 onwards). Under these growth conditions, *LACK*/*LACK^DDE^* L. major also showed a polysome profile with multiple polysome peaks. Unexpectedly, similar to WT, LACK^DDE^ also cosedimented with the ribosomal marker P0 in monosome/polysome fractions (Fig. 4B). These data indicate LACK^DDE^ associates efficiently with *Leishmania* ribosomes at 27°C.

We also observed unexpected differences between the polysome profile trace of *LACK*/*LACK^DDE^* and *LACK*/*LACK^RDG^* L. major (Fig. 4A and B, upper panels). First, the overall abundance of the 40S, 60S, 80S, and polysome peaks was lower in *LACK*/*LACK^DDE^* than *LACK*/*LACK^RDG^ L. major*. Second, the abundance ratio of 60S to 40S subunits was lower for *LACK*/*LACK^DDE^ L. major*. Third, a shoulder was observed at the base of the monosome, disome, and trisome peaks (see arrows in Fig. 4B). The latter observation indicates the presence of polysome half-mers in the *LACK/LACK^DDE^* line and is consistent with the loss of 60S relative to 40S subunits. Polysome half-mers are polysomes bearing an extra 40S subunit and occur when the relative abundance of the 60S subunits becomes limited (17–19). Despite these perturbations, both *LACK*/*LACK^RDG^* and *LACK*/*LACK^DDE^* lines showed comparable growth kinetics under these conditions (Fig. 3A).

**FIG 5:**
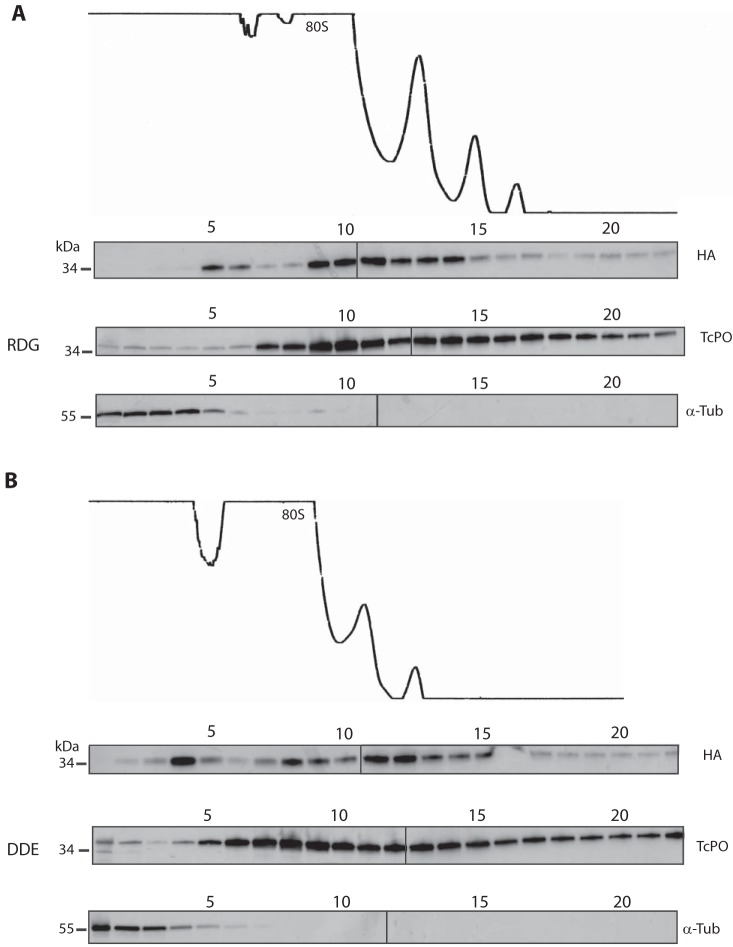
*LACK^DDE^* associates with polysomes at 35°C. Polysome profiles from *LACK*/*LACK^RDG^* (RDG) and *LACK*/*LACK^DDE^* (DDE) L. major cultures, established as described in Materials and Methods, are shown as described in the legend to Fig. 4. The fractions were probed with anti-HA, anti-TcP0, and anti-α-tubulin antisera, as indicated. “80S” denotes 80S monosomes. Fraction numbers are indicated above each blot. The data shown are from one experiment representative of two independent clones, each performed in duplicate. Protein size markers (kDa) are indicated at left of each panel.

The robust association of LACK^DDE^ with *Leishmania* polysomes at 27°C was unexpected, based on the findings of others for S. cerevisiae (8) and T. brucei RACK1 proteins (L. Ruben, Southern Methodist University, Dallas, TX, unpublished data). However, since *LACK*/*LACK^DDE^* L. major showed no fitness defects at this temperature, we reasoned that this was consistent with LACK^DDE^’s ability to associate with ribosomes under these conditions. At 35°C, however, we speculated that we would observe decreased ribosomal association of LACK^DDE^ because of the *LACK*/*LACK^DDE^* fitness defects (Fig. 3B) observed under these conditions. We therefore performed polysome analyses of *LACK*/*LACK^RDG^* and *LACK*/*LACK^DDE^* lines cultured at 35°C. Surprisingly, immunoblot analyses of the *LACK*/*LACK^RDG^* and *LACK*/*LACK^DDE^* lines revealed that both LACK^RDG^ and LACK^DDE^ cosedimented efficiently with ribosomes and polysomes at this elevated temperature (Fig. 5).

Given our unexpected results, we further confirmed the integrity of our 35°C polysome preparations by analyzing the distribution of α-tubulin, a protein that does not associate with polysomes (20). Indeed, in both lines, α-tubulin did not associate with the P0 ribosomal marker (Fig. 5A and B, lower panels), similar to previous polysome analyses in other trypanosomatids (20). Although immunoblot analyses identified no major differences in LACK^DDE^ cosedimentation with P0 and polysomes, we did observe a minor shift of LACK^DDE^ into the mRNP fractions. This coincided with a minor shift away from the monosome fractions, compared to LACK^RDG^ (compare Fig. 5A and B). Regardless of these latter observations, our findings indicate that even under conditions that expose the *LACK*-deficient phenotype of *LACK*/*LACK^DDE^* L. major, LACK^DDE^ remains largely able to associate robustly with actively translating ribosomes. Therefore, in our experiments with L. major, LACK^DDE^ behaves differently from DDE mutants of the canonical RACK1^RDK^ proteins studied in other eukaryotes.

**FIG 6:**
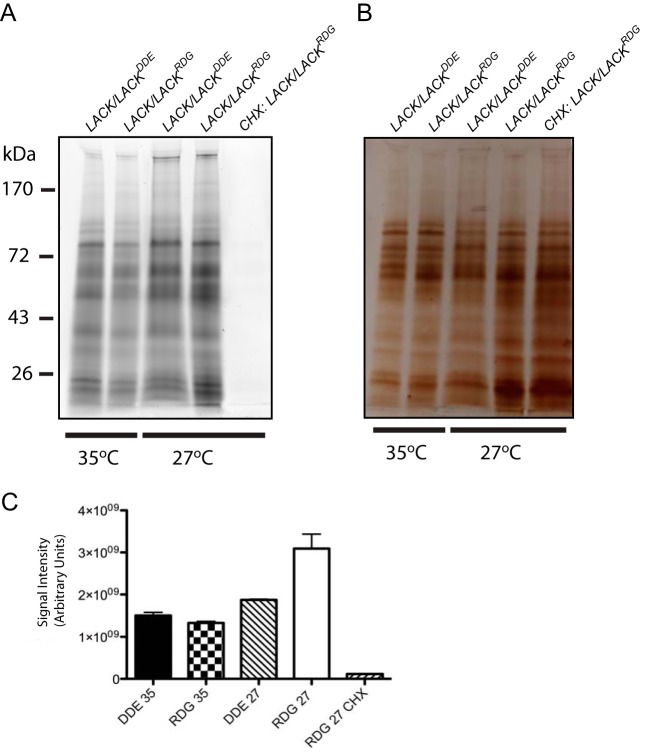
*LACK*/*LACK^DDE^* L. major show robust nascent global protein synthesis at 27 and 35°C. Logarithmically growing parasites were established at 27 and 35°C, as described in Fig. 4 and 5, respectively. (A) After 3 h of incubation with azidohomoalanine (AHA) in methionine-free RPMI 1640 medium–10% dialyzed FBS, parasites were lysed, and nascent protein synthesis was detected via click-chemistry coupling to TAMRA, followed by SDS-PAGE electrophoresis and imaging using a Typhoon TRIO+ variable mode Imager (GE) with a 532-nm excitation and 580-nm 30-nm band pass filter. As a negative control, we also included lysate from parasites that were treated with cycloheximide (CHX) immediately prior to addition of AHA and incubation for 3 h. The image shown is representative of two independent experiments, each performed with a different clone of each line, as indicated. (B) After imaging for nascent protein synthesis, the total protein present in each lane was assessed by silver staining. The data shown are from one experiment, representative of two independent experiments, each with different clones, performed in duplicate. (C) Average total imaged band intensities in each lane were quantified from the two independent experiments performed in duplicate, using Typhoon/Molecular Dynamics analysis software (GE). Protein size markers (kDa) are indicated.

When cultured at 35°C, the height and number of polysome peaks were decreased in both the *LACK*/*LACK^RDG^* and *LACK*/*LACK^DDE^* lines (Fig. 5A and B, upper panels). Conversely, the abundance of 40S and 60S subunits and 80S monosomes was increased. Although LACK^RDG^ and LACK^DDE^ both cosedimented with *Leishmania* ribosomes and polysomes at 35°C, we did observe distinct polysome traces for *LACK*/*LACK^DDE^* and *LACK*/*LACK^RDG^* L. major (Fig. 5A and B, upper panels). The *LACK*/*LACK^DDE^* line showed moderately decreased polysome peaks (disomes and trisomes) compared to the *LACK*/*LACK^RDG^* line (disomes, trisomes and tetrasomes). Interestingly, although half-mers were observed at 27°C (Fig. 4), they were not detected at 35°C (Fig. 5). Previous studies (21, 22) demonstrated that *Leishmania* undergoes a heat shock response when culture temperature is elevated to 35°C. Interestingly, unlike other *Leishmania* species, global translation in L. major is not substantially decreased under these conditions (22). Considering the altered polysome profiles and thermosensitivity observed for *LACK*/*LACK^DDE^* L. major (Fig. 4 and 5), we sought to determine whether these phenotypes correlated with global alterations in protein synthesis. Using azidohomoalanine labeling, we found nascent global protein synthesis was robust, ranging from approximately 1.5 × 10^9^ to 3 × 10^9^ arbitrary units of averaged image signal intensity in both lines at 27 and 35°C (Fig. 6A and C). At 27°C, averaged nascent global protein synthesis was highest for *LACK*/*LACK^RDG^* L. major; however, nascent protein syntheses were comparable in both lines at 35°C. Conversely, in the presence of the protein synthesis inhibitor cycloheximide, no products were detected (Fig. 6A, 5th lane), confirming detection of nascent protein synthesis in lanes 1 to 4. Total protein in each lane was confirmed by silver staining the gel after imaging (Fig. 6B). Therefore, unexpectedly, global protein synthesis in *LACK*/*LACK^DDE^* L. major was relatively efficient under both conditions despite altered polysome profiles.

### At 35°C, expression of LmCOX4 and LmCOX6 is abrogated in *LACK*/*LACK^DDE^ L. major*.

**FIG 7:**
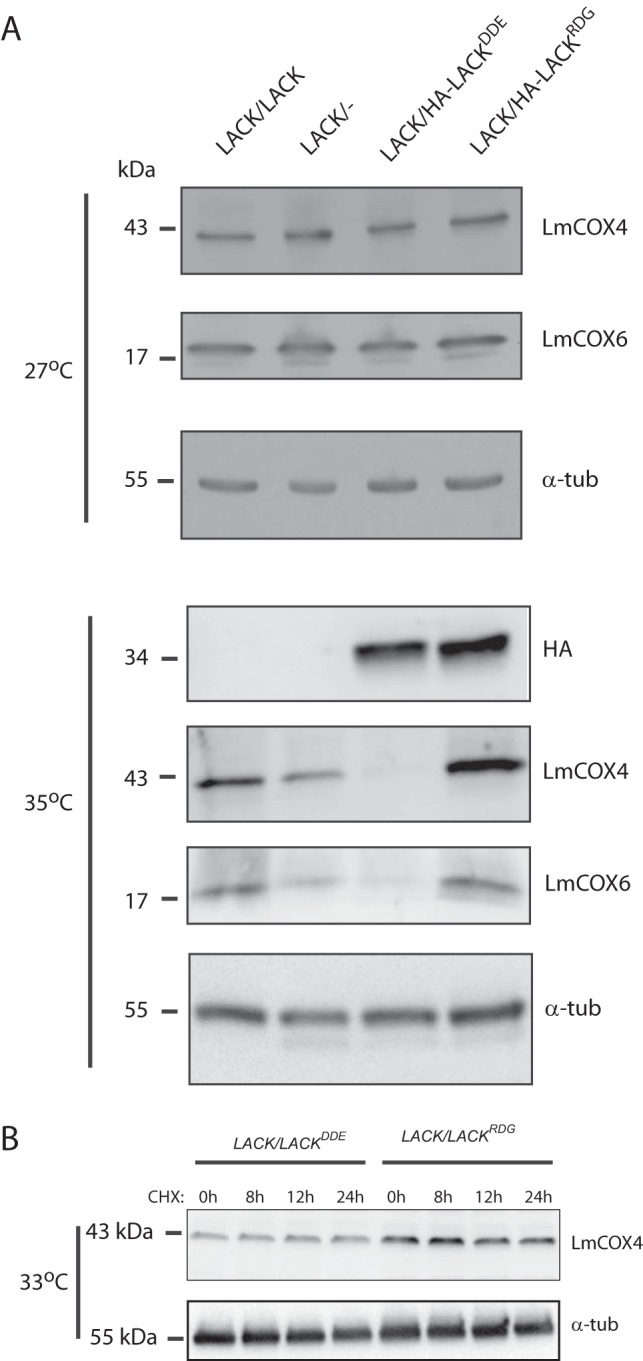
*LACK*/*LACK^DDE^* L. major exhibits decreased levels of LmCOX4 and LmCOX6 at mammalian temperature. (A) Immunoblot analysis of lysates obtained from the indicated L. major lines grown for 5 days at 27 or 35°C, as indicated. The blot was probed with anti-LmCOX4 or anti-LmCOX6 antiserum (raised against *T. brucei* COVI [6]) and then stripped and reprobed with anti-α-tubulin antibody. (B) Parasites were incubated as described for panel A, except that the incubation temperature was 33°C instead of 35°C. Cycloheximide was added to a final concentration of 70 μg/ml, and the parasites were incubated at 33°C for an additional 24 h. Aliquots of approximately 4 × 10^7^ parasites were removed at 8, 12, and 24 h and processed for immunoblot analysis, as indicated. “0h” denotes removal of aliquots immediately prior to cycloheximide addition. The blot was probed with anti-LmCOX4, stripped, and then reprobed with anti-α-tubulin, as indicated. The data shown are from one experiment, representative of two or three independent clones, performed as two to four replicates. Protein size markers (kDa) are indicated to the left of each panel.

We next investigated whether the *LACK*/*LACK^DDE^* mutant displayed LmCOX4 expression defects similar to those of *LACK*/− L. major. We performed immunoblotting on lysates from parasite lines grown at both 27 and 35°C to determine the levels of LmCOX4 and LmCOX6 proteins. At 27°C, LmCOX4 and LmCOX6 levels were alike in all four L. major lines (Fig. 7A, upper panel), similar to previous findings for *LACK*/*LACK* and *LACK*/− L. major (6). Conversely, at 35°C, the amount of LmCOX4 protein was dramatically reduced in *LACK*/*LACK^DDE^* L. major (Fig. 7A, lower panel). Interestingly, while LmCOX4 levels in *LACK*/− L. major were significantly lower than in the *LACK*/*LACK* and *HA-LACK*/*LACK^RDG^* lines, they were almost undetectable in the *LACK*/*LACK^DDE^* line. Thus, LmCOX4 expression in *LACK*/*LACK^DDE^* L. major was more dramatically impaired than in the *LACK*/− strain. Consistent with our previous studies suggesting LmCOX6 expression may be dependent upon LmCOX4 (6), we also saw loss of LmCOX6 expression in *LACK*/*LACK^DDE^* L. major under these conditions (Fig. 7A, lower panel).

To investigate mechanisms underlying the decreased LmCOX4 levels in *LACK*/*LACK^DDE^* L. major, we examined whether LmCOX4 had a shorter half-life in *LACK*/*LACK^DDE^* L. major by treating the parasites with cycloheximide (Fig. 7B). At 35°C, the almost undetectable levels of LmCOX4 make it difficult to assess potential decay of LmCOX4 in *LACK*/*LACK^DDE^* L. major. Therefore, we incubated the parasites at 33°C instead. At this temperature, although LmCOX4 levels remain substantially decreased in *LACK*/*LACK^DDE^* L. major, they remain sufficiently abundant to detect by Western blotting. As shown in Fig. 7B, we found that exposure to cycloheximide over 24 h caused no loss of LmCOX4 in either *LACK*/*LACK^DDE^* or *LACK*/*LACK^RDG^* L. major, relative to their respective untreated controls. These data suggest that decreased protein half-life does not contribute to the loss of LmCOX4 observed in *LACK*/*LACK^DDE^* L. major at elevated temperature.

### *LACK*/*LACK^DDE^ L. major* has impaired mitochondrial function.

**FIG 8:**
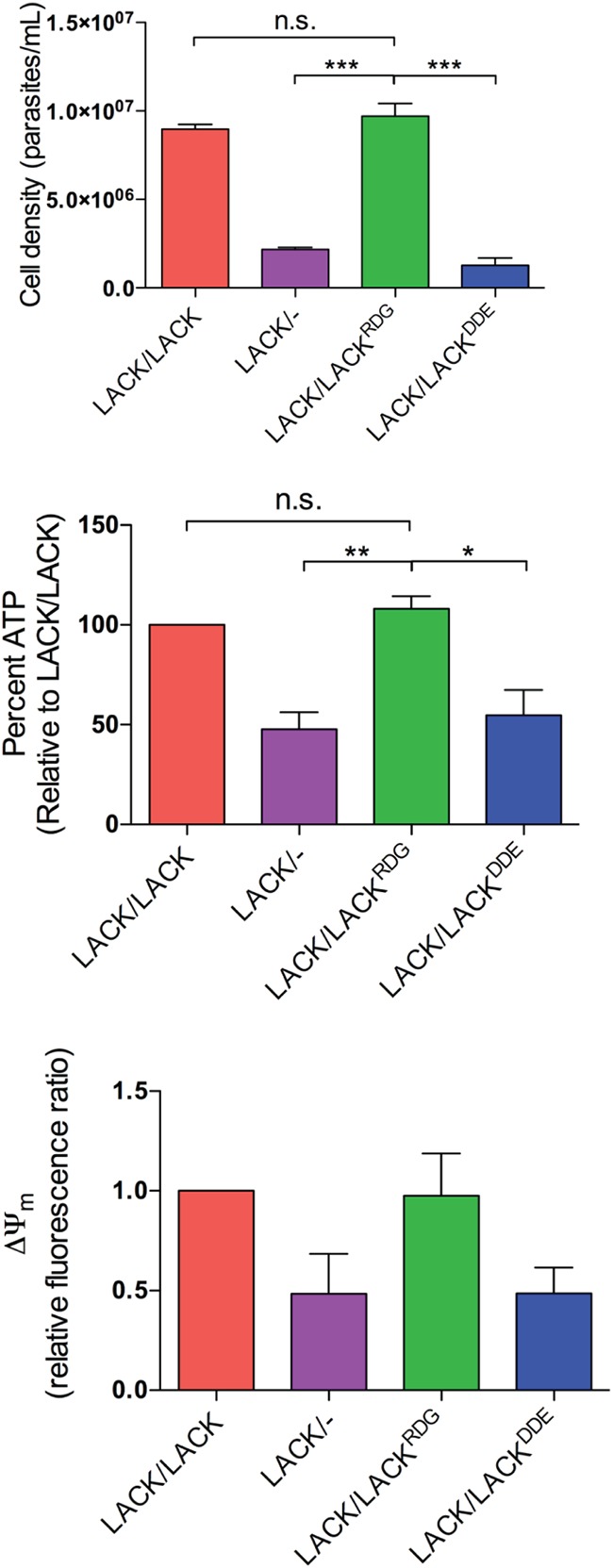
The *LACK*/*LACK^DDE^* line displays attenuated mitochondrial function at 35°C. For each line indicated, cell population density was determined after incubation at 35°C for 5 days as noted in Fig. 3B. For each line, ATP levels and mitochondrial membrane potential (ΔΨm) were determined from 1 × 10^6^ parasites. Briefly, ATP was quantified by luminometer using the Cell Titer-Glo reagent and mitochondrial membrane potentials were measured by plate reader, using the fluorescent probe JC-1, as previously described (6). Data are shown as means ± SEM from two independent experiments, each using a different clone of each line, performed as three to four replicates.

To examine the functional impact of replacing an endogenous *LACK* copy with *LACK^DDE^* upon parasite mitochondrion function, we assayed for ATP levels and mitochondrial membrane potential after 5 days of culture at 35°C. At this time point, the *LACK*/*LACK^DDE^* line exhibited a drastic decrease in cell population density compared to *LACK*/*LACK^RDG^* L. major (Fig. 8, top panel). To assess the mitochondrial phenotype, all lines were normalized for cell number prior to assaying. Compared to virulent controls, there was a substantial decrease in ATP levels and mitochondrial membrane potential in the *LACK*/*LACK^DDE^* line, comparable to that in *LACK*/− L. major (Fig. 8, middle and bottom panels). These data indicate that, unlike targeted replacement of the downstream endogenous *LACK* ORF with *HA-LACK^RDG^*, replacement with *HA-LACK^DDE^* results in mitochondrial dysfunction.

### *LACK*/*LACK^DDE^ L. major* shows attenuated virulence in macrophages.

**FIG 9:**
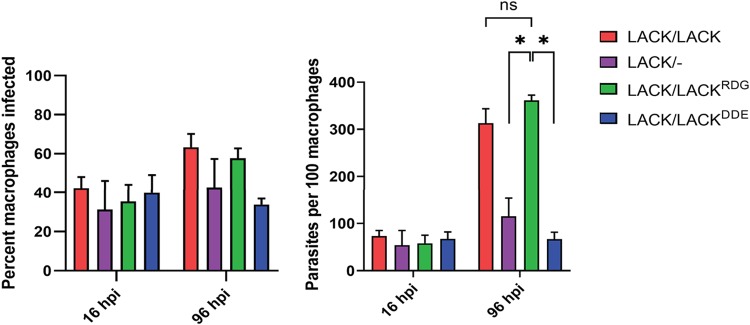
*LACK*/*LACK^DDE^* exhibits attenuated virulence in macrophages. A total of 5 × 10^5^ primary murine macrophages were incubated overnight in chamber slides at 35°C and 5% CO_2_. Macrophages were then infected with 5 × 10^6^ stationary-phase promastigotes from the indicated L. major lines for 4 h, after which noninternalized promastigotes were removed by washing. The infection was allowed to proceed for 16 or 96 h, as indicated, before fixing and staining the cells with Hoechst 33342. For each L. major cell line, the percentage of macrophages infected and number of parasites per 100 macrophages were determined, as indicated. Data are shown as means ± SEM from over 20 random fields from two independent experiments, each using an independent clone of each L. major line indicated, performed in quadruplicate. *P* values were determined by the two-tailed Student's *t* test. A result of *P* < 0.05 was considered statistically significant and is denoted with an asterisk.

Previously, we determined that *LACK*/− parasites replicate less efficiently in macrophages compared to *LACK*/*LACK* parasites (5, 6). Thus, we assessed whether *LACK*/*LACK^DDE^* L. major also shows attenuated macrophage parasitization. We infected macrophages at a multiplicity of infection (MOI) of 10 and allowed the infection to proceed for 4 h prior to washing off unbound parasites. After 16 or 96 h, the slides were fixed and stained with Hoechst 33342. These time points were chosen to distinguish between parasite entry and establishment in macrophages (16 h postinfection) versus replication within macrophages (96 h postinfection). For each parasite line, the infections were scored by fluorescence microscopic enumeration of parasite nuclei per 100 macrophage nuclei and percentage of macrophages infected as previously described (6). At 16 h postinfection, there were no significant differences between any of the parasite lines (Fig. 9, left panel). However, at 96 h postinfection, although the trends for percentage of infected macrophages were not found to be statistically significant (Fig. 9, left panel), *LACK*/*LACK^DDE^* L. major exhibited significantly decreased parasites/100 macrophages compared to *LACK*/*LACK^RDG^* parasites (Fig. 9, right panel). As expected, the infective phenotype of the *LACK*/*LACK^RDG^* line was similar to that of *LACK*/*LACK* L. major at 96 h postinfection (6). These results demonstrate that, similar to *LACK*/− L. major, *LACK*/*LACK^DDE^* L. major has an attenuated virulence phenotype; the parasites enter macrophages with similar efficiency to the virulent *LACK*/*LACK* and *LACK*/*LACK^RDG^* lines, but replicate poorly in the phagolysosome.

## DISCUSSION

LACK is a ribosome-associated RACK1 ortholog important for *Leishmania* fitness and virulence in the vertebrate host (5). At least two *LACK* gene copies are required for parasite mitochondrial fitness and virulence (5, 6). Recently, we identified the mitochondrial *L. major* cytochrome *c* oxidase subunit, LmCOX4, as a novel molecular target for LACK since under mammalian conditions, normal LmCOX4 expression levels depend upon two *LACK* copies (6). Significantly, LACK’s role in parasite fitness and virulence has been attributed to its ability to sustain LmCOX4 expression under mammalian conditions (6).

RACK1 family members are multifunctional scaffold proteins, involved in spatial control of a variety of cellular pathways, including kinase signaling. They are also important in translation, acting via the ribosome to promote expression of specific proteins (10, 23, 24). Consistent with a translational role for LACK, earlier studies demonstrated it is a functional constituent of *Leishmania* ribosomes, involved in translation initiation (7).

To elucidate molecular mechanisms underlying LACK-dependent LmCOX4 expression, we investigated whether LACK promotes LmCOX4 expression through its putative ribosome-associated functions. Previous studies indicated that disruption of RACK1-ribosome association could be achieved by substitution of RACK1’s canonical RDK motif with a DDE sequence. This approach has been used to investigate RACK1’s translation-specific functions (8, 10, 15, 25). Similar to earlier studies of RACK1 (8, 26), analysis of LACK’s interaction with *Leishmania* ribosomes (13) predicts that replacement of LACK’s noncanonical orthologous motif, RDG, with DDE will disrupt LACK’s ribosomal interaction. We therefore generated an L. major line in which one of the two endogenous *LACK* copies required for parasite thermotolerance was replaced with *LACK^DDE^*, an HA-tagged copy of *LACK* encoding the D_34_D_35_E_36_ mutation. Our finding that at 27°C the *LACK^DDE^* variant cosedimented efficiently with L. major polysomes was unexpected. Interestingly, using a chemical cross-linking approach to study ASC1, the S. cerevisiae RACK1 ortholog, Thompson et al. (15) showed that the *ASC1^DDE^* mutant line (ASC1^DE^) grows normally and ASC1^DE^ associates with polysomes *in vivo*. These studies corroborate our findings with *LACK*/*LACK^DDE^* L. major cultured at 27°C.

Our studies also revealed unexpected differences between the S. cerevisiae ASC1^DE^ mutants and *LACK*/*LACK^DDE^* L. major. First, while protein cross-linking was required to demonstrate *in vivo* ribosomal association of ASC1^DE^, this step was not necessary in our study. These data suggest that association between RACK1 orthologs and ribosomes may be stronger in L. major than S. cerevisiae. Second, we found that despite normal growth at 27°C, *LACK*/*LACK^DDE^* L. major showed alterations in ribosomal status. These included relative loss of 60S subunits, an observation further confirmed by the appearance of ribosome half-mers in the monosome and polysome fractions. Thompson et al. (15) also observed depletion of the 60S subunit relative to 40S ribosomal subunit. However, unlike our study, this was only in the slowly growing ASC1 null mutants, not the ASC1^DE^ mutant which grew normally at 30°C.

The decreased 60S ribosomal subunit abundance observed for *LACK*/*LACK^DDE^* L. major suggests LACK itself may contribute to 60S ribosomal subunit biogenesis. For example, LACK may facilitate the expression of 60S ribosomal subunit proteins. The latter possibility is consistent with studies of Schizosaccharomyces pombe showing that expression of the 60S ribosomal subunit protein rpL25 is RACK1 dependent (24). The altered ribosomal status of *LACK*/*LACK^DDE^* L. major correlated with moderately decreased global protein synthesis. This suggests a minor impairment of translational efficiency under these conditions. Regardless, the normal 27°C growth phenotype of *LACK*/*LACK^DDE^* L. major and the robust global production of nascent proteins indicate that such decreases in these protein-synthetic components are generally well tolerated in this line. This growth phenotype is also similar to that of attenuated *LACK*/− L. major at 27°C (6). Only upon exposure to mammalian environmental conditions, such as elevating temperature to 35°C, or during macrophage infection were the defects in LmCOX4 expression and mitochondrial fitness exposed in these two lines. Interestingly, at 35°C, the LmCOX4 expression defect in *LACK*/*LACK^DDE^* L. major was even more pronounced than that in the *LACK*/− line. These data suggest LACK^DDE^ may be interfering with the function of endogenous WT LACK remaining in the *LACK*/*LACK^DDE^* L. major line.

Despite the decreases in cell growth, polysomes and LmCOX4 expression at 35°C, metabolic labeling confirmed global protein synthesis in *LACK*/*LACK^DDE^* L. major was unimpaired. Interestingly, recent studies indicate that RACK1^DDE^ remains functional with respect to global translation (27) and 80S monosomes are translationally active (28). It is possible that WT LACK is acting at the ribosome via its RDG motif and promoting translation of specific mRNAs; these could include those encoding LmCOX4 itself or a modulator of LmCOX4 expression. This scenario is consistent with recent studies suggesting that RACK1 is indeed required for efficient translation of specific mRNAs (27).

Although at 35°C, the polysome peaks were diminished in *LACK*/*LACK^DDE^* L. major, we did not observe the polysome half-mers that were present at 27°C. Given the lower abundance of the disome and trisome peaks in this line under these conditions, it is possible that any associated half-mer populations may be below the level of detection.

Unexpectedly, despite the striking 35°C phenotypic defects of *LACK*/*LACK^DDE^* L. major, LACK^DDE^ associated efficiently with *Leishmania* polysomes. As discussed earlier, these observations contrast with conventional polysome studies of RACK1^DDE^ mutants, including those in the closely related trypanosomatid T. brucei RACK1 (8; L. Ruben, unpublished data). The altered polysome profiles of *LACK*/*LACK^DDE^* L. major at 27 and 35°C, however, are consistent with a disruptive effect of LACK^DDE^ on the ribosome. This phenotype may be a consequence of suboptimal positioning of LACK^DDE^ on the ribosome. Indeed, this possibility is corroborated by recent studies, suggesting that although ASC1^DE^ mutants can associate with the ribosome *in vivo*, their exact positioning on the 40S subunit may be distorted (29, 30). Thus, our data suggest RACK1^DDE^ variants may have impaired functions despite their ribosomal association.

We cannot exclude the possibility that LACK^DDE^ associates with ribosomes at both 27 and 35°C because of structural differences between LACK and other RACK1 orthologs. We therefore considered the possibility that the interaction between LACK and *Leishmania* ribosomes is structurally distinct from RACK1^RDK^, perhaps involving sites other than the RDG motif. If this hypothesis is correct, we could expect compensatory structural differences in the ribosomes to accommodate LACK binding. It would therefore not be unreasonable to expect that such differences may be incompatible with canonical RACK1^RDK^ function. This scenario seems unlikely, however, since transgenic expression of TbRACK1, the T. brucei RACK1^RDK^, functionally substitutes for LACK and TbRACK1 cosediments efficiently with *Leishmania* ribosomes (7).

Because RACK1s have been shown to form homodimers (31, 32), we also considered whether, in *LACK*/*LACK^DDE^* L. major, the endogenous WT LACK remaining in this line dimerizes with LACK^DDE^. Thus, as a putative LACK^RDG^/LACK^DDE^ heterodimer, LACK^DDE^ may associate with the ribosome indirectly, via the RDG motif of its WT dimerization partner. This scenario could also potentially explain the more severe LACK-deficient phenotype of *LACK*/*LACK^DDE^* compared to *LACK*/− L. major, where in the latter, all putative dimers would be LACK/LACK homodimers. However, structural studies indicate that RACK1 proteins interact with *Leishmania* ribosomes with a 1:1 stoichiometry (13), arguing against the formation of LACK homodimers at the ribosome.

In this study, we investigated molecular mechanisms that underlie LACK-dependent expression of L. major LmCOX subunits in the hostile environment of mammalian phagocytes. Expression of LmCOX subunits is critical for parasite mitochondrial function, supporting fitness and hence virulence in the vertebrate host (6). We sought to determine whether LACK promotes LmCOX subunit expression via its interaction with the ribosome (7). We therefore replaced one of the two endogenous *LACK* copies required for virulence with *LACK^DDE^*. *LACK^DDE^* encodes a predicted ribosome-binding-defective variant of LACK. Surprisingly, LACK^DDE^ associated robustly with both monosomes and polysomes. Nonetheless, although no major global translational defects were observed, we found temperature-condition-specific impairment of LmCOX subunit expression. Our findings suggest that in *LACK*/*LACK^DDE^* L. major, LACK^DDE^ may be positioned on the ribosome in a conformation that is deleterious to LmCOX4 expression. Furthermore, the decreased levels of LmCOX4 appear not to be a result of accelerated protein decay. Thus, we speculate that despite retaining its ability to associate with ribosomes, LACK^DDE^, unlike WT LACK, does not support efficient translation of LmCOX4. Interestingly, such defects only impact LmCOX subunit expression at mammalian temperature. Molecular mechanisms to further explain the link between replacement of WT *LACK* with the *LACK^DDE^* variant and severely impaired LmCOX subunit expression will be pursued in the future.

Our observations indicate that certain properties of LACK are shared with its orthologs in other species. However, we have also identified interactions between LACK and ribosomes in L. major that are distinct from those in other eukaryotes. This study further highlights the unique biology of *Leishmania* and diversity of RACK1 interactions across species. Such differences may potentially be exploited for development of novel antiparasitic therapies.

## MATERIALS AND METHODS

### Parasites.

All L. major lines used were derived from WT L. major strain WHOM/IR/-/173 and cultured at 27 or 35°C in M199 with 10% heat-inactivated fetal bovine serum (FBS), as previously described (5). For cycloheximide treatment, parasites were incubated at 33°C for 8, 12, and 24 h in 70 μg/ml cycloheximide as previously described (33).

### Construction of plasmids and generation of *LACK2*-targeted lines encoding HA-tagged LACK mutants.

Point mutations in the *LACK2* ORF were introduced by PCR-based site-directed mutagenesis. The fragment of the *LACK* ORF upstream of the predicted ribosome-binding motif codons (minus the *LACK* start codon) was amplified from WT L. major genomic DNA by PCR using a PacI restriction enzyme site-tagged forward primer and a reverse primer containing the desired mutation to the putative ribosome-binding motif codons. The fragment of the *LACK* ORF downstream of the putative ribosome-binding motif codons was amplified from WT L. major genomic DNA by PCR using a forward primer containing the desired mutation to the putative ribosome-binding motif codons and a reverse primer containing a PacI restriction enzyme site. The two *LACK* fragments were then fused by PCR using the same PacI restriction enzyme site-tagged forward and reverse primers described above to produce the full-length *LACK* ORF encoding a mutation in the putative ribosome-binding motif. All primers used are annotated in Table 1. This fragment was cloned into the pGEM-T Easy vector (Promega, Madison, WI) and submitted for sequencing (ACGT, Inc., Wheeling, IL). Following verification of the correct sequence, the insert was excised from pGEM-T Easy with PacI and ligated downstream of the 3× HA tag in PacI-cleaved pL2PD-HALK-L2, encoding a marker conferring resistance to puromycin (previously described [7]). As previously indicated, the downstream *LACK* gene (*LACK2*) from *LACK*/*LACK* L. major was replaced by transfection with linearized inserts (7) gel purified from the constructs described above containing either the *HA-LACK^RDG^* or *HA-LACK^DDE^* ORF, giving rise to the *LACK*/*LACK^RDG^* and *LACK*/*LACK^DDE^* L. major lines, respectively. These lines express one endogenous copy of *LACK* and one HA-tagged copy of *LACK* containing either the WT putative ribosome-binding motif (RDG) or mutated version (DDE), as indicated (Fig. 2). Clones of each line were isolated after plating on semisolid M199 containing 22 µg ml^−1^ puromycin (5). Faithful gene targeting in each clone was confirmed by Southern blot analysis. The *LACK*/*LACK* and *LACK*/− L. major lines were described previously (6).

**TABLE 1:** Primers used for the construction of HA-LACK^RDG^- and HA-LACK^DDE^-targeting plasmids^*a*^

ORF	PacI-LACK forward primer	RBM mutation reverse primer	RBM mutation forward primer	PacI-LACK reverse primer
*HA-LACK^RDG^*	5′-CTCTTAATTAAGAACT ACGAGGGTCACCTGAAG-3′			5′-CTCTTAATTAAGTTAC TCGGCGTCGGAGATGG-3′
*HA-LACK^DDE^*	5′-CTCTTAATTAAGAACT ACGAGGGTCACCTGAAG-3′	5′-GTCTCATCATCGACGT CGACACCACCTTG-3′	5′-GATGATGAGACGGCCA TCTCGTGGAAGG-3'	5′-CTCTTAATTAAGTTAC TCGGCGTCGGAGATGG-3′

a The PacI restriction enzyme site is underlined for the PacI-LACK forward and reverse primers. The resulting ribosome-binding motif (RBM) mutation, DDE, is underlined for the RBM mutation forward and reverse primers.

### Southern blot analysis.

Genomic DNA from the virulent *LACK*/*LACK* L. major control line as well as the *LACK*/*LACK^RDG^* and *LACK*/*LACK^DDE^* lines was extracted with phenol-chloroform (34), digested overnight with StuI, concentrated by ethanol precipitation, and size separated by agarose gel electrophoresis. The DNA was transferred onto Hybond-N+ nylon membrane (GE Healthcare Life Sciences, Piscataway, NJ) by alkali capillary blotting following the manufacturer’s instructions. A biotin-labeled probe, derived from the *LACK* ORF, was synthesized using a NEBlot Phototope kit (New England BioLabs, Ipswich, MA) and purified using a QIAquick nucleotide removal kit (Qiagen, Venlo, The Netherlands) according to the manufacturer’s recommendations. The labeled probe was hybridized to the target DNA at 65°C using Rapid-hyb buffer (GE Healthcare Life Sciences) according to the manufacturer’s instructions. Following hybridization, the membrane was washed in 2× saline sodium citrate (SSC) plus 0.5% SDS followed by 0.2× SSC plus 0.5% SDS as previously reported (7). The blot was visualized by chemiluminescence using the Phototope-Star detection kit (New England BioLabs) as per the manufacturer’s specifications.

### Immunoblot analyses.

A total of 0.5 × 10^7^ to 1 × 10^7^ parasites were washed with ice-cold phosphate-buffered saline (PBS) and resuspended in ice-cold radioimmunoprecipitation assay (RIPA) buffer or Laemmli buffer containing 1 mM phenylmethylsulfonyl fluoride (PMSF), 1 µg ml^−1^ pepstatin A, 1 µg ml^−1^ leupeptin, and 1 µg ml^−1^ aprotinin. After incubation on ice for 30 min, lysates were further disrupted by being passaged 6 times through a 27-gauge needle. The lysates were clarified by centrifugation at 12,000 × *g* for 15 min at 4°C, and the protein concentration was determined by bicinchoninic acid (BCA) assay (Pierce, Rockford, IL). Clarified lysates were resuspended in sample buffer (50 mM Tris-HCl [pH 6.8], 2% SDS, 10% glycerol, 1% β-mercaptoethanol, 12.5 mM EDTA, 0.02% bromophenol blue) and heated to 95°C for 5 min. Proteins were separated by SDS-PAGE on 12% polyacrylamide gels and transferred onto polyvinylidene difluoride (PVDF) membranes (Bio-Rad, Hercules, CA) in accordance with the manufacturer’s instructions. Membranes were blocked for 1 h at room temperature in 3% blotting-grade blocker (Bio-Rad, CA) or 5% nonfat dry milk in 0.05% PBS-Tween 20 (PBS-T). Membranes were then washed in PBS-T and probed overnight at 4°C with 1:1,500 rat anti-HA (clone 3F10) or 1:2,000 mouse anti-α-tubulin (clone DM1A). We generated anti-LmCOX4 antisera by raising antisera against recombinant LmCOX4, expressed and purified as follows. The LmCOX4 ORF was inserted into a pET15 expression vector containing a C-terminal hexahistidine tag and then transformed into E. coli Rosetta2 (Novagen) competent cells. Protein expression was induced by the addition of 1 mM isopropyl β-d-thiogalactopyranoside (IPTG). Overnight postinduction cells were collected by centrifugation and resuspended in binding buffer (50 mM HEPES [pH 7.5], 500 mM NaCl, 1 mM benzamidine, and 1 mM phenylmethanesulfonyl fluoride [PMSF]). The cells were lysed by adding chemical lysis buffer consisting of 2% CHAPS {3-[(3-cholamidopropyl)-dimethylammonio]-1-propanesulfonate}, 0.1 mg/ml lysozyme, and 0.5 U/ml Benzonase, and the lysate was clarified by centrifugation. Inclusion bodies were dissolved in 50 mM HEPES (pH 7.5) and 8 M urea. Solubilized proteins were loaded on to a nickel-nitrilotriacetic acid column preequilibrated with denaturing binding buffer (50 mM HEPES [pH 7.5], 8 M urea). Following a wash step with denaturing binding buffer containing 5 mM imidazole, the bound protein was eluted with elution buffer (50 mM HEPES [pH 7.5], 8 M urea, 250 mM imidazole). Purified proteins were dialyzed against decreasing concentrations of urea (from 4 to 2 to 1 M) in PBS. The protein purity was evaluated by SDS-PAGE gels and quantified using the BCA assay (Pierce). The recombinant LmCOX4 protein was used to immunize rabbits according to standard protocols (Pocono Rabbit Farm and Laboratory, Inc., Canadensis, PA). Anti-LmCOX4 was used at 1:2,500. After washing in PBS-T, blots were incubated with either 1:6,000 goat anti-rat Ig, 1:3,000 goat anti-rabbit Ig, or 1:6,000 goat anti-mouse Ig conjugated with horseradish peroxidase for 1 h at room temperature. The blots were washed in PBS-T and developed using ECL enhanced chemiluminescence reagents (GE Healthcare Life Sciences) according to the manufacturer's instructions.

### Growth assays.

Parasites were incubated at either 27 or 35°C in M199 at a starting concentration of 5 × 10^5^ parasites ml^−1^, unless otherwise specified. Each culture was enumerated daily by taking an aliquot, fixing the parasites in 0.4% formaldehyde in PBS, and counting using a hemocytometer and an Eclipse Ti-S inverted microscope (Nikon, Tokyo, Japan).

### Polysome analyses.

Sucrose gradient fractionation of *Leishmania* polysomes was performed essentially as described previously (14). Briefly, 5 × 10^8^ to 7.5 × 10^8^ cells were obtained from logarithmically growing cells cultured at 27°C in M199 with 10% FBS, as previously described (6); cultures were seeded with late-logarithmic-phase parasites at a density of ∼2 × 10^6^ cells/ml. The cells were cultured for 1.5 days and then harvested. The harvested cells were resuspended in 5 ml of M199–10% FBS containing cycloheximide (200 µg/ml) for 10 min at 27°C. For cells cultured at 35°C, late-logarithmic-phase parasites were set up at a density of 2 × 10^6^ cells/ml, incubated at 35°C overnight, and then diluted 1 in 2.5 with fresh medium and then incubated at 35°C for another 24 h. The cells were then harvested and incubated with cycloheximide as described for the 27°C cultures. The cells were then incubated on ice for 5 min prior to being pelleted by centrifugation. The cells were then washed in 50 ml ice-cold TKM buffer (15 mM Tris-HCl [pH 7.4], 0.3 M KCl, 5 mM MgCl_2_) containing 200 µg/ml cycloheximide. The parasites were resuspended in 900 µl TKM buffer containing 0.5 mM DTT, 200 µg/ml cycloheximide, 1 mg/ml heparin, 10 µM E64 (cysteine protease inhibitor), and 1:100 EDTA-free protease inhibitor cocktail (Roche). The cells were then lysed by adding 100 µl of TKM lysis buffer (TKM buffer, 10% IGEPAL CA630) and then pipetting the mixture up and down 20 times with a P1000 pipette tip. Lysates were then centrifuged at 16,000 × *g* for 15 min at 4°C. Lysate supernatants were then layered onto 10 to 50% sucrose linear gradients made up in TKM buffer, supplemented with 200 µg/ml cycloheximide, 1 mg/ml heparin, and 10 µM E64. The gradients were centrifuged for 120 min at 35,000 rpm using an SW-41 Ti rotor (Beckman Instruments). Fractions of 0.5 ml each were then collected from the top of the gradient using a Foxy Jr. fraction collector (Teledyne Isco, Inc.). Proteins were concentrated from each fraction by methanol-chloroform precipitation as described earlier (7). The proteins were dissolved in sample buffer and submitted to Western blot analysis, using rat anti-HA antibody 3F10 (1:1,500), rabbit anti-TcP0 (1:2,500), and mouse anti-α-tubulin (1:2,000).

### Assessment of nascent protein synthesis.

Nascent protein synthesis was assessed in 2-day logarithmically growing cultures at 27 and 35°C as previously described (35). Briefly, the parasites were washed in PBS and then resuspended in methionine-free RPMI 1640 medium (Gibco) containing 10% dialyzed FBS and incubated for 1 h at 27 or 35°C. l-Azidohomoalanine (Invitrogen) was then added to a final concentration of 50 μM in accordance with the manufacturer’s instructions, and then the cells were labeled by incubation for 3 h at 27 or 35°C. The parasites were then lysed in 1% SDS–50 mM Tris-HCl containing 250 U/ml Benzonase (EMD Millipore). Protein concentrations for each sample were determined by BCA assay, and then the proteins were reacted with the Click-iT-TAMRA-alkyne (Invitrogen) detection reagent in accordance with the manufacturer’s instructions. After SDS-PAGE, the size-fractionated TAMRA (6-carboxytetramethylrhodamine)-labeled nascent proteins were imaged using a Typhoon TRIO+ variable-mode fluorescent imager (GE Healthcare).

### Mitochondrial membrane potential assay.

Mitochondrial membrane potentials were assessed by incubating the parasites in medium 199 (10% FBS) at 27 or 35°C, using the fluorescent probe JC-1 (Cayman Chemical, Ann Arbor, MI), in accordance with the manufacturer’s instructions.

### Determination of ATP levels.

L. major ATP levels were quantified by luminometer using the Cell Titer-Glo (Promega, Madison, WI) according to the manufacturer’s instructions, as described previously (6).

### Macrophage infections.

Macrophages were isolated from previously euthanized mice kindly provided by Matthew Whim (Department of Cell Biology and Anatomy, Louisiana State University Health Sciences Center [LSUHSC]) according to a protocol approved by the LSUHSC Institutional Animal Care and Use Committee (IACUC) under IACUC protocol no. 3134.

A total of 5 × 10^5^ peritoneal macrophages, isolated from C57BL/6 mice by peritoneal lavage, were incubated for 24 h at 35°C and 5% CO_2_ in RPMI 1640 medium supplemented with 5% FBS, 100 U ml^−1^ penicillin, 100 µg ml^−1^ streptomycin, 2 mM l-glutamine, and 2 mM nonessential amino acids using Nunc Lab-Tek II chamber slides (Thermo Fisher Scientific, Waltham, MA). Nonadherent cells were removed from each chamber by washing with warm (37°C) PBS. Stationary-phase promastigotes were added to the macrophages at a multiplicity of infection (MOI) of 10 (5 × 10^6^ parasites) and incubated for 4 h at 35°C and 5% CO_2_. After the incubation, parasites that had not been internalized were removed by washing three times with warm PBS. After addition of 0.8 ml of fresh RPMI 1640 to each chamber, the infection was allowed to proceed for either 16 or 96 h.

At the end of the infection time point, the slides were washed three times in PBS and sequentially incubated in 4% paraformaldehyde for 20 min, 0.1% Triton X-100 for 4 min, and 1:3,000 diluted Hoechst 33342 in PBS for 2 min. (Slides were washed twice with PBS between each step.) The slides were mounted using Fluoromount-G mounting medium (Southern Biotech, Birmingham, AL). Macrophages and parasites were detected using an Axio Observer.Z1 fluorescence microscope (Carl Zeiss AG, Oberkochen, Germany) with a 63× objective. The infection burden of the macrophages was measured by enumerating intracellular parasite nuclei per at least 300 macrophage nuclei from over 20 random fields.

### Statistical analysis.

*P* values were determined by the two-tailed Student's *t* test, using Prism software (GraphPad, La Jolla, CA). A result of *P* < 0.05 was considered significant and is denoted with an asterisk.
