# Supplementary material for: Disruption of the Putative Ribosome-Binding Motif of a Scaffold Protein Impairs Cytochrome c Oxidase Subunit Expression in Leishmania major
Source: mSphere. 2019 Mar 6;4(2):e00644-18. doi: 10.1128/mSphere.00644-18 (PMC6403457; doi:10.1128/mSphere.00644-18)
Supplement: FIG S1 [file mSphere.00644-18-sf001.pdf]

S1

```
LmLACK       YEGHLKGHRGWVTSLACPQQAGSYIKVVSTSRDGTAISWKANPDRHSVDSDYGLPNHRLE  62
             YEG L GHRGWVTSLACPQ   +  KVVSTSRD T +SW  NPDRHS +  YGLP+ RLE
TbRACK1      YEGQLTGHRGWVTSLACPQTPETATKVVSTSRDKTLLSWGPNPDRHSSECSYGLPDRRLE  64

LmLACK       GHTGFVSCVSLAHATDYALTASWDRSIRMWDLRNGQCQRKFLKHTKDVLAVAFSPDDRLI  122
             GH+ FVS V+L++  ++A++ASWD S+R+W+L+NGQCQ KFL HTKDVL+VAFSPD+R I
TbRACK1      GHSAFVSDVALSNNGNFAVSASWDHSLRLWNLQNGQCQYKFLGHTKDVLSVAFSPDNRQI  124

LmLACK       VSAGRDNVIRVWNVAGECMHEFLRDGHEDWVSSICFSPSLEHPIVVSGSWDNTIKVWNVN  182
             VS GRDN +RVWNV GECMH   R  H DWVS + FSPSL+ P++VSG WDN +KVW++
TbRACK1      VSGGRDNALRVWNVKGECMHTLSRGAHTDWVSCVRFSPSLDAPVIVSGGWDNLVKVWDLA  184

LmLACK       GGKCERTLKGHSNYVSTVTVSPDGSLCASGGKDGAALLWDLSTGEQLFKINVESPINQIA  242
              G+     LKGH+NYV++VTVSPDGSLCAS  KDG A LWDL+ GE L ++   +PINQI
TbRACK1      TGRLVTDLKGHTNYVTSVTVSPDGSLCASSDKDGVARLWDLTKGEALSEMAAGAPINQIC  244

LmLACK       FSPNRFWMCVATERSLSVYDLESKAVIAELTPD--GAKP--SECISIAWSADGNTLYSGH  298
             FSPNR+WMC ATE+ + ++DLE+K +I EL P+  G+K    EC+SIAWSADG+TLYSG+
TbRACK1      FSPNRYWMCAATEKGIRIFDLENKDIIVELAPEHQGSKKIVPECVSIAWSADGSTLYSGY  304

LmLACK       KDNLIRVWSISD  310
              DN+IRVW +S+
TbRACK1      TDNVIRVWGVSE  316
```

https://blast.ncbi.nlm.nih.gov/Blast.cgi?PAGE=Proteins&PROGRAM=blastp&BLAST_PROGRAMS=blastp&PAGE_TYPE=BlastSearch&BLAST_SPEC=blast2seq
