# Supplementary figures and images for: Disruption of the Putative Ribosome-Binding Motif of a Scaffold Protein Impairs Cytochrome c Oxidase Subunit Expression in Leishmania major
Source: mSphere. 2019 Mar 6;4(2):e00644-18. doi: 10.1128/mSphere.00644-18 (PMC6403457; doi:10.1128/mSphere.00644-18)

S2

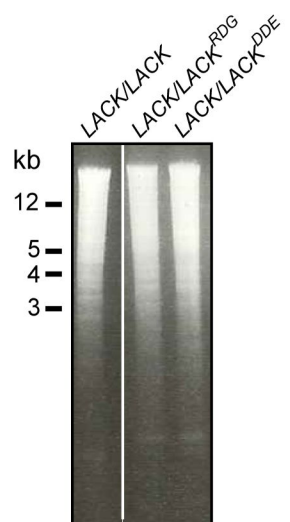

Supplement: FIG S2 [file mSphere.00644-18-sf002.pdf]

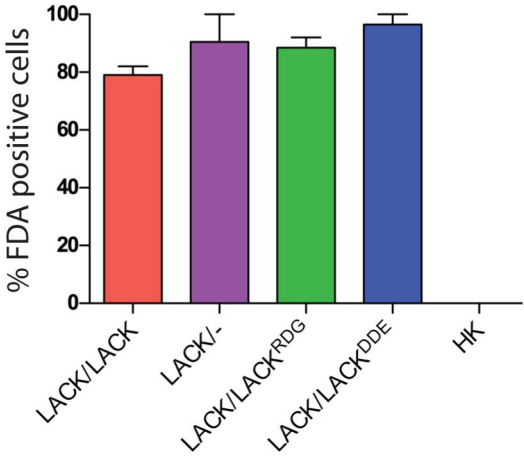

Supplement: FIG S3 [file mSphere.00644-18-sf003.pdf]
